# Interrater Reliability of the Spanish (Colombia) Version of the Post-COVID-19 Functional Status Scale

**DOI:** 10.1155/2023/1124661

**Published:** 2023-11-09

**Authors:** Vicente Benavides-Córdoba, Juan Carlos Ávila-Valencia, Diana Guerrero-Jaramillo, Luz Alejandra Lorca, Mauricio Palacios, Rodrigo Torres-Castro, Jhonatan Betancourt-Peña

**Affiliations:** ^1^Universidad del Valle, Cali, Colombia; ^2^Institución Universitaria Escuela Nacional del Deporte, Clínica de Occidente S.A.S., Cali, Colombia; ^3^Secretaria Distrital de Salud, Cali, Colombia; ^4^Hospital del Salvador, Santiago, Chile; ^5^Department of Physical Therapy, University of Chile, Santiago, Chile; ^6^Institución Universitaria Escuela Nacional del Deporte, Universidad del Valle, Cali, Colombia

## Abstract

**Background:**

COVID-19 has been one of the most critical public health challenges of recent decades. This disease develops severely in one in five patients, and approximately 5% require admission to a critical care unit. Due to the impact of the sequelae, the Post-COVID-19 Functional Status Scale (PCFS) was developed. This study is aimed at determining the interrater reliability of the Spanish (Colombia) version of the PCFS in adult patients with post-COVID-19 infection.

**Methods:**

This is an observational study performed with patients diagnosed with COVID-19. Two evaluators repeated the test-retest of the PCFS scale with knowledge and clinical experience in the care of patients with COVID-19 and had previously applied the test. The PCFS assesses functional limitations at discharge and can be used at 4 and 8 weeks to evaluate practical consequences and determine the degree of disability these patients may have. For interrater reliability, Cronbach's alpha was applied with its respective confidence interval and the Bland-Altman method. A 95% confidence interval (CI) was taken as the basis for the interpretation of the Intraclass Correlation Coefficient (ICC).

**Results:**

A total of 112 adult patients participated in the study, aged 51.46 ± 15.94 years. It was evidenced that the survival, constant care, and activities of daily living questions have an ICC of one (1.000) with an ICC (1.000-1.000), demonstrating excellent reliability, while those close to one were instrumental activities, role participation, symptoms, and final score with an ICC 0.918 to 0.984 and an ICC (0.881-0.989). Thus, a homogeneous distribution of the interrater data was evident.

**Conclusions:**

Excellent interobserver reliability of the Spanish (Colombia) version of the PCFS in patients with different degrees of functional status was reported.

## 1. Introduction

COVID-19 has been one of the most critical public health challenges of recent decades [1]. This disease develops severely in one in five patients, and approximately 5% require admission to a critical care unit [2]. In addition, those who survive a critical illness are likely to develop medium- and long-term sequelae that affect their functioning and quality of life [3].

The literature presents numerous physical, respiratory, cardiovascular, and mental health sequelae [4, 5]. Although the disease primarily affects the respiratory system, it can affect other cardiovascular or neurological systems. Populations at the most significant risk of developing severe disease are those with comorbidities and advanced age [6–8].

Recent experience with COVID-19 has highlighted the need for a multidisciplinary approach to follow-up, especially in patients with advanced age, obesity, comorbidities, and organ failure [9, 10]. In addition, a follow-up of patients with post-COVID-19 sequelae should include a comprehensive evaluation that consists of a specific assessment focused on respiratory, cognitive, physical, and functional limitations [10].

Concerning functional limitations, a significant impact of the disease on the adequate performance and independence of activities of daily living has been identified, with the appearance of disability and reduced quality of life, with impairments that can persist for up to 1 to 6 months [11], for which scales such as the Barthel scale have frequently been used [12]. An adequate understanding of what is happening at the functional level in patients is essential for making clinical and epidemiological intervention decisions, and also because COVID-19 develops medium- and long-term sequelae, such as cognitive and physical sequelae, that affect patient's functioning and quality of life, the rehabilitation interventions are fully considered to improve the functional status of the patient.

Due to the impact of sequelae, the Post-COVID-19 Functional Status Scale (PCFS) [13] and the study of Machado which reported the construct validity of the scale and the strongest association with the “usual activities” domain of the 5-level EQ-5D questionnaire [13].

Currently, the PCFS manual and instructions are available in several translations through their website (https://osf.io/qgpdv/(CC-BY 4.0)). In addition, some translated versions have validation or psychometric measures [14–17].

A reliable scale must be used in the clinical or research setting [18, 19]. An unreliable tool cannot yield consistent results, so it is impossible to determine whether scores are due to actual differences or measurement errors [20]. One method to assess reliability is to perform an interrater reliability study. This study measures the agreement between different raters in assessing the same phenomenon using the same test [19]. Therefore, this study is aimed at determining the interrater reliability of the Spanish (Colombia) version of the PCFS in adult patients with post-COVID-19 infection.

## 2. Methods

### 2.1. Study Design and Population

This study is a prospective observational study and is subject to the recommendations of the *Strengthening the Reporting of Observational Studies in Epidemiology* (STROBE) [21].

### 2.2. Participants

Adult patients were diagnosed with COVID-19 who were required with in-hospital management between March and December 2021 in Cali, Colombia, for a minimum of 10 days and who had already been discharged from hospitalization (*n*: 112). Patients who did not agree to participate in the study, who had been hospitalized for complications other than COVID-19, who did not have the mental conditions to answer the scale, who were minors, and who did not speak Spanish were excluded.

Clinical history data supplemented the information, and the patients who agreed to participate in the study signed the informed consent. The ethics committee of the *Clínica de Occidente* endorsed the project ID: IYECDO-1261.

### 2.3. Variables and Procedures

#### 2.3.1. Information Collection

Data were collected directly from a patient interview. The diagnosis of COVID-19 was confirmed with a reverse transcription-polymerase chain reaction (RT-PCR) test [22]. Data were collected on age, sex, socioeconomic status, marital status, occupation, days of hospitalization, and history, including comorbidities such as diabetes, arterial hypertension, cardiovascular disease, and pulmonary disease. Likewise, relevant data were collected during hospitalization, such as the need for supplemental oxygen and intensive care unit (ICU) stay. Dyspnea was assessed using the mMRC scale [23].

Evaluators who independently applied the scale randomly assigned patients using Microsoft Excel® 2021 (Redmond, Washington, USA). In turn, the investigators were masked during the study.

### 2.4. Reliability

#### 2.4.1. Test-Retest

Two evaluators repeated the test-retest of the PCFS scale with knowledge and clinical experience in the care of patients with COVID-19 and who had previously applied the test. In addition, a pilot test was performed with 10% of the total sample. These pilot test data were not included in the reliability analysis. The scale was applied with an interval of 3 days between each evaluator.

### 2.5. Outcomes

#### 2.5.1. PCFS

The PCFS assesses functional limitations at discharge and can be used at four and eight weeks and six months after discharge to consider the practical consequences and determine the degree of disability these patients may have. The PCFS score ranges from 0 to 5, with 0 indicating no functional limitation and 5 signifying death [13].

The PCFS stratifies patients' functional conditions, starting from 0, where there are no practical limitations; grade 1, where there are negligible functional limitations; grade 2, slight functional limitations; grade 3, moderate functional limitations; grade 4, severe functional limitations; and classification D (death). The scale has two application forms: a structured interview and a self-application. In this study, the structured interview was applied since it facilitates the objective assigned to the grades of the scale. The categories of survival, constant care, basic activities of daily living (BADLs), instrumental activities of daily living (iADLs), participation in usual social roles, and symptom checklist were included [13].

### 2.6. Statistical Analysis

Statistical analysis was performed with GraphPad Prism version 8.0 (GraphPad Software, San Diego, CA, USA). Quantitative variables are presented as the mean ± standard deviation, and qualitative variables as frequencies and percentages. The chi^2^ test and Spearman correlations allowed comparing the ratings by each evaluator for the PCFS scale domains; they were classified considering three categories: poor (*r* ≤ 0.49), moderate (0.50 ≤ *r* ≤ 0.74), and strong (*r* ≥ 0.75).

For interrater reliability, Cronbach's alpha with its respective confidence interval and the Bland-Altman method was applied to measure the degree of agreement between two quantitative variables. Additionally, the agreement between the two measurements conducted by the evaluators was assessed using a 95% confidence interval (CI) as the basis for interpreting the intraclass correlation coefficient (ICC). Acceptable ICC values greater than 0.7 were categorized as follows: excellent (≥0.9), good (<0.9 to ≥0.8), and acceptable (<0.8 to ≥0.7). The primary objective of calculating the ICC in this study was to assess the consistency and reliability of measurements taken by different raters or at different time points, thus ensuring the accuracy and robustness of our results.

## 3. Results

**Figure 1:**
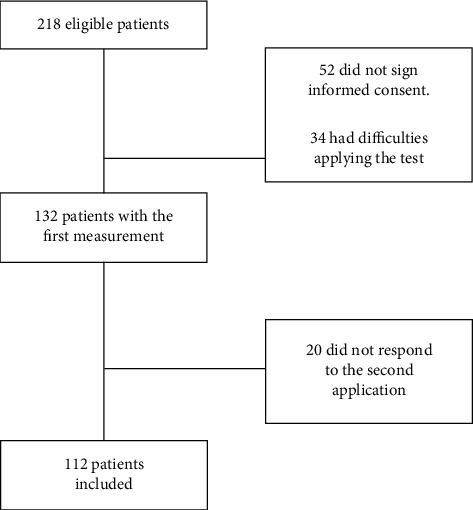
Admission of COVID-19 patients.

The findings show that out of 218 patients, 52 did not accept the informed consent; 34 had difficulties applying the test, and 20 did not respond to the second application. Finally, 112 adult patients participated in the study (Figure 1), mainly women (69, corresponding to 61.6%), with a mean age of 51.46 ± 15.94 (Table 1).

Regarding the patients' clinical conditions, the mean mMRC dyspnea was 1.28 ± 1.19. Regarding comorbidities, 47 (42%) were overweight/obese; 31 (27.7%) had hypertension; 20 (17.9%) had diabetes; 9 (8.0%) had cardiovascular or pulmonary disease; 3 (2.7%) had hypothyroidism. Most participants were hospitalized in an intensive care unit *n* = 80 (71.4%) and had a mean number of hospital days of 19.5 ± 9.5. In turn, 103 (92.0%) patients required supplemental oxygen during hospitalization, of whom the majority, 72 (64.3%) of them, required supplemental oxygen after hospital discharge (Table 1).

The predominant risk factor was a sedentary lifestyle with 68 (60.7%) patients. Table 1 shows the results of the two evaluators on the scale regarding the SCAF classification. It was evidenced that more patients were classified with a score of 2 by both evaluators, being statistically significant.

Regarding the correlations by domains in the PCFS scale, between the evaluators, it was found that for all parts, strong correlations *r* ≥ 0.75 were presented, being the constant care and ADL environments the ones that offered the best correlations with values of 1.000 (Table 2). In addition, differences in each of the domains between raters could not be calculated for the variables of survival, constant care, and ADLs since the mean values and standard deviations were equal. However, in the domains of iADL, participation, symptoms, and final score, there were no statistically significant differences between the groups, corroborating the similar results presented by both evaluators (Table 3).

Values of one and close to one were obtained for the ICC. This fact demonstrated excellent interrater agreement reliability when applying the scale. It was evident that the survival, constant care, and activities of daily living questions have an ICC of one (1,000) with a 95% confidence interval of (1,000-1,000); this demonstrates excellent reliability. Meanwhile, those close to one were instrumental activities, role participation, symptoms, and final score with an ICC of 0.918 to 0.984 and a 95% confidence interval of (0.881-0.989); this demonstrates good reliability (Table 4).

**Figure 2:**
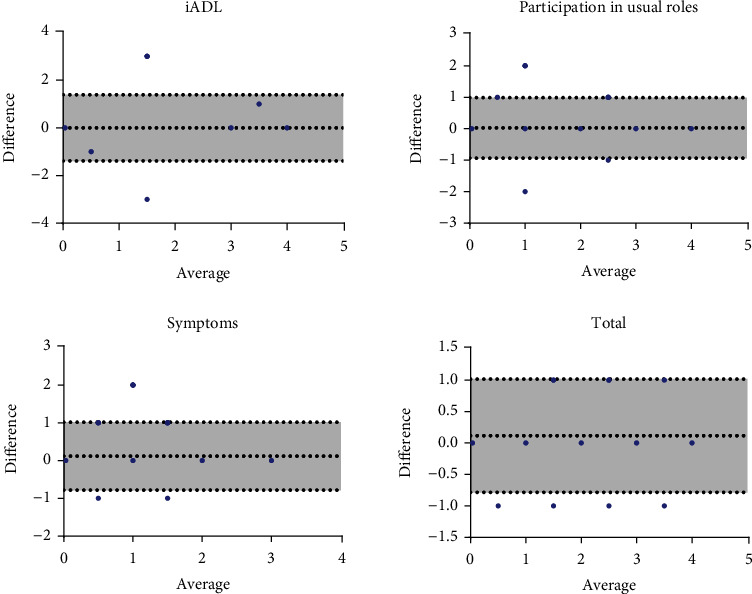
Bland-Altman method. Calculation of the 95% CI of the bias and the limits of agreement.

Homogeneous distribution of the interrater concordance data was evidenced, most of them within two standard deviations and located at the zero line (0), such as the variables of survival, constant care, and basic activities of daily living (ADL). In instrumental activities (Figure 2), most of the data were found within the standard deviations 1.392-1,392, close to the zero line with a mean difference of zero, in role participation (Figure 2). Most of the data are close to the zero line between standard deviations of 0.9384-0.9920, with a mean difference of 0.02, for symptoms (Figure 2).

## 4. Discussion

This study is aimed at determining the interrater reliability of the PCFS scale, Spanish version (Colombia), in adult patients with post-COVID-19 infection. The primary outcome is that the PCFS scale showed excellent interrater reliability. This result was obtained in patients hospitalized for a minimum period of 10 days. This decision was made because the disease causes varying degrees of severity [2, 24].. To obtain variable results in the assessment of functionality, it was decided to consider only those patients with the highest risk of functional compromise.

Reliability is the ability of an instrument to measure consistently [25]. The importance of making these measurements lies in obtaining reliable instruments that allow relevant results to be obtained with a good level of certainty, regardless of the original language. This is because these studies allow for multilingual adaptation after translation and cultural adaptation, as has been applied with other tools for different diseases [26]. Cronbach's alpha score is higher in patients with complex severity levels. However, reliability remains very high in patients without symptoms. Likewise, the degree of agreement between the variables evaluated, measured with the Bland-Altman test, was very high, similar to that obtained with the reliability tests. These results are comparable with a previous study conducted with a Spanish version, and the PCFS scale showed adequate construct validity and provided substantial test-retest reliability (kappa = 0.63). Furthermore, a strong correlation has been observed with multiple questionnaires, including the Short Form-36, Hospital Anxiety and Depression Scale, modified Medical Research Council, and Borg Six-Minute Walk Test [27, 28].

The authors of the PCFS scale reported that it could be used for postdischarge functional assessments and long-term evaluations [13]. Moreover, it is an easy-to-use tool that can be applied in low-resource settings for a follow-up and rehabilitation programs [29, 30]. For this reason, multiple translations have been made worldwide, allowing large-scale applications. However, in the case of this study, specifically in Colombian patients, it is striking that 92% of the participants required supplemental oxygen during hospitalization, and more than 60% required it even after hospital discharge.

Hypoxic pulmonary vasoconstriction is a well-identified phenomenon. Clinical observations have been made in patients with severe COVID-19 [31]. It has been identified as marked hypoxemia, accompanied by high-grade infiltrates, pulmonary vascular endothelialitis, and microthrombus formation [32, 33], which further compromises the patient's condition. This may also be related to the levels of functional dyspnea obtained with the mMRC scale and the number of patients presenting with a scale score of 4, showing marked functional limitations. Also, a relationship between PCFS and functional dyspnea has been identified [17]. In a study performed in 121 patients, they identified a high correlation with the mMRC dyspnea scale, finding a rho = 0.53 (*p* < 0.0001) [28]. The relationship between the two scales lies in their common goal of assessing dyspnea and functionality in patients with respiratory disease. The association between the mMRC score and the PCFS score can be related with that close to 60% of post-COVID-19 patients that have effort dyspnea during daily life, limiting the functional performance [13]. While the mMRC has been widely used as a standard measure in this field, the PCFS scale complements this evaluation and provides a more specific and focused assessment in the context of COVID-19 patients.

Regarding other translations of the scale, the Turkish version obtained similar results to those obtained in this investigation, with excellent reliability; the Cronbach's alpha value was 0.82 in the total score, having a CI of 0.734 and 0.880 [17]. This is comparable with the results obtained by our research in which the value of the final Cronbach's alpha score was 0.98 with a CI of 0.977-0.989, both translations being in the good/excellent range. Additionally, in another article that evaluated the Brazilian Portuguese version of the PCFS scale, the reliability was moderate to excellent. This version was administered by health professionals as an online structured interview via video-conferencing platform to patients with post-COVID-19, after hospital discharge [34]. This finding implies that the translated versions of the PCFS are reliable and can be administered in varying severity. The inclusion of patients who required ICU management is an essential difference between the two studies, as these patients were included in our study. COVID-19 disease can manifest with a wide range of severity, from mild cases to those requiring intensive care. We believe that including patients who required ICU management favors a complete understanding of the impact of the disease on the functionality of the affected individuals [17].

Other versions of the scale have been evaluated; the Spanish version from Spain showed good convergent validity demonstrating significant relationships between patients' functional status, quality of life, limitations of activities of daily living, and psychological status, specifically showing that an increase in functional status was associated with improvement in the other variables [27]. In Netherlands and Belgium, in a study conducted with the original language version of the PCFS, the authors identified weak to strong associations between functional status and several quality of life scales, demonstrating the strongest association with the “usual activities” domain in the 5-level version of the EQ-5D questionnaire [15].

It is essential to recognize that other functionality assessment scales, such as the Barthel scale, have also been used in other chronic diseases. Researchers have also used it in COVID-19 with good results [35].

The study's main limitation is the difficulty of reaching a large sample. This is due to the same inclusion criteria since several patients were excluded because they mainly needed to complete the required days of hospitalization. However, this situation can also be taken as a point in favor of the heterogeneity of the PCFS groups. Also, from self-criticism, high Cronbach's alpha scores could mean redundancy in some items [36]. However, interrater applications allow us to infer that no matter who applies the test, the result will be the same.

## 5. Conclusions

Excellent interrater reliability of the Spanish (Colombia) version of the Post-COVID-19 Functional Status Scale was presented in patients with different degrees of functional condition. It is necessary to continue applying the scale in the hospital and outpatient context and the follow-up of patients.

## Figures and Tables

**(a):** 

Variables	*n* = 112
Age (years)	51.46 ± 15.94^∗^
Gender	
Female	69 (61.6%)
Male	43 (38.4%)
Socioeconomic status	
Low	32 (37.5%)
Medium	66 (58.9%)
High	4 (3.6%)
Marital status	
Married	47 (41.9%)
Single	65 (58.1%)
Occupation	
Healthcare professional	29 (25.9%)
Others	17 (15.2%)
Home	12 (10.7%)
Independent	12 (10.7%)
Unemployed	10 (8.9%)
Retired	8 (7.1%)
Transporter	7 (6.3%)
Administrative worker	5 (4.5%)
Comorbidities	
Diabetes	20 (17.9%)
Arterial hypertension	31 (27.7%)
Cardiovascular disease	9 (8.0%)
Lung disease	9 (8.0%)
Hypothyroidism	3 (2.7%)
Type of hospitalization	
Hospitalized	32 (28.6%)
Days hospitalized^∗^	18.9 ± 10.8^∗^
Intensive care unit	80 (71.4)
Days ICU^∗^	19.5 ± 9.5^∗^
Oxygen during hospitalization	103 (92.0%)
Oxygen after discharge	72 (64.3%)
Risk factors	
Obesity-overweight	47 (42.0%)
Smoke (last month)	13 (11.6%)
Alcohol (last month)	10 (8.9%)
Sedentary lifestyle	68 (60.7%)

**(b):** 

PCFS Scale grade^∗∗^	Evaluator 1	Evaluator 2
PCFS 0	21 (18.7%)	20 (18.8%)
PCFS 1	21 (18.7%)	22 (19.6%)
PCFS 2	35 (31.3%)	34 (30.3%)
PCFS 3	17 (15.2%)	18 (16.1%)
PCFS 4	18 (16.1%)	18 (16.1%)

^∗^Values are expressed as the mean and standard deviation. ^∗∗^Chi^2^ test between PCFS degrees (*p* value < 0.001). PCFS: Post-COVID-19 Functional Status Scale.

**Table 2:** Correlations between the PCFS variables.

Variable (PCFS)	Rho	*p* value
Survival evaluator 1/evaluator 2	—	—
Constant care evaluator 1/evaluator 2	1.000	<0.001
ADL evaluator 1/evaluator 2	1.000	<0.001
IADL evaluator 1/evaluator 2	0.889	<0.001
Participation Evaluator1/Evaluator 2	0.931	<0.001
Symptoms evaluator 1/evaluator 2	0.851	<0.001
Final score evaluator 1/evaluator 2	0.966	<0.001

ADL: basic activities of daily living; IADL: instrumental activities.

**Table 3:** Differences of PCFS.

Variables	Mean difference	SD	95% limits of agreement
Survival	—	—	—
Constant care	—	—	—
ADL	—	—	—
IADL^∗^	0.00	0.70	1.39–1.39
Participation^∗^	0.02	0.49	0.93–0.99
Symptoms^∗^	0.11	0.46	0.78–1.0
Final score^∗^	-0.01	0.32	0.66–0.62

^∗^ *p* value ≥ 0.05. ADL: basic activities of daily living; IADL: instrumental activities.

**Table 4:** Intraclass correlation coefficient.

Variable (PCFS)	ICC	95% CI	Alfa de Cronbach
Survival	1.000	1.000–1.000	1.000
Constant care	1.000	1.000–1.000	1.000
ADL	1.000	1.000–1.000	1.000
IADL	0.948	0.925–0.965	0.948
Participation	0.965	0.950–0.976	0.965
Symptoms	0.918	0.881–0.944	0.918
Final score	0.984	0.977–0.989	0.984

ICC: intraclass correlation coefficient; CI: confidence interval; ADL: basic activities of daily living; IADL: instrumental activities.

## Data Availability

Data is available at doi:10.6084/m9.figshare.21725300.

## Additional Points

*Highlights*. Patients with COVID-19 presented excellent interrater reliability on the PCFS scale. The PCFS scale may be helpful for healthcare professionals in Colombia. The PCFS scale can be used for postdischarge functional and long-term assessments in patients with COVID-19.
